# ESFT13: A Phase II Study Evaluating the Addition of Window and Maintenance Therapy to a Standard Chemotherapy Backbone for the Treatment of High-Risk Ewing Sarcoma

**DOI:** 10.3390/cancers17172894

**Published:** 2025-09-03

**Authors:** Jessica Gartrell, Fariba Navid, Xiaomeng Yuan, Kirsten K. Ness, Mikhail Dubrovin, Fang Wang, Haitao Pan, Mary Beth McCarville, Barry L. Shulkin, Sara Helmig, Matthew J. Krasin, Michael D. Neel, Andrew M. Davidoff, Belinda N. Mandrell, Deena R. Levine, Zhongheng Cai, Michael W. Bishop, Alberto S. Pappo, Sara M. Federico

**Affiliations:** 1 Department of Oncology, St. Jude Children’s Research Hospital, Memphis, TN 38105, USAsara.federico@stjude.org (S.M.F.); 2 Cancer and Blood Disease Institute, Children’s Hospital Los Angeles, Department of Pediatrics, Keck School of Medicine, University of Southern California, Los Angeles, CA 90027, USA; fnavid@chla.usc.edu; 3 Department of Biostatistics, St. Jude Children’s Research Hospital, Memphis, TN 38105, USAhaitao.pan@stjude.org (H.P.);; 4 Department of Epidemiology and Cancer Control, St. Jude Children’s Research Hospital, Memphis, TN 38105, USA; 5 Department of Radiology, Columbia University, New York, NY 10032, USA; 6 Department of Radiology, St. Jude Children’s Research Hospital, Memphis, TN 38105, USAbarry.shulkin@stjude.org (B.L.S.); 7 Department of Radiation Oncology, St. Jude Children’s Research Hospital, Memphis, TN 38105, USA; matthew.krasin@stjude.org; 8 Department of Surgery, St. Jude Children’s Research Hospital, Memphis, TN 38105, USA; 9 Department of Pediatric Medicine, St. Jude Children’s Research Hospital, Memphis, TN 38105, USA; 10 Department of Pediatric Hematology-Oncology, Arkansas Children’s Hospital, Little Rock, AR 72202, USA

**Keywords:** Ewing sarcoma, pediatrics, irinotecan, temozolomide, temsirolimus, maintenance chemotherapy

## Abstract

Study Aim: Patients with high-risk Ewing sarcoma (ES) have dismal outcomes despite aggressive multimodal therapy. This phase II, single-institution study evaluated the response rate to two up-front cycles of irinotecan, temozolomide, and temsirolimus (ITT) and assessed the tolerability of maintenance therapy following standard treatment in high-risk ES. Methods: Eligible patients had newly diagnosed high-risk ES (age ≥14 years old, metastatic disease, or primary pelvic tumor). The therapy included two cycles of window therapy (ITT) followed by interval-compressed chemotherapy (vincristine, doxorubicin, and cyclophosphamide alternating with ifosfamide and etoposide) and maintenance therapy (cyclophosphamide, sorafenib, and bevacizumab). A two-stage sequential design was employed to assess a >50% WHO response (CR or PR) with 80% power. Patients who required emergent radiation were excluded from receiving window therapy. Results: Sixteen patients (median age 12.2 years; range 4.8–23.6 years) were enrolled (12 evaluable for overall response, 10 for primary tumor response). Only three achieved a PR to window therapy, leading to study closure. All evaluable patients demonstrated a decline in their primary tumor volume (mean decline: 32.5%, standard deviation: 17.6%, *p*-value: 0.0005) and SUV peak (mean decline: 49.9%, standard deviation: 21.1%, *p*-value: 0.002). Maintenance therapy was well tolerated, with only 2/13 patients discontinuing due to toxicity. Conclusions: ITT did not achieve the prespecified response rate of 50%, according to WHO criteria; however, all patients exhibited decreased volume and metabolic activity, highlighting the limitations of conventional response assessments. Maintenance therapy was feasible and well tolerated. Although limited by small sample size, heterogeneous disease presentations, and the absence of a control arm, this study supports further evaluation of ITT and a maintenance approach in larger, randomized trials for high-risk ES.

## Simple Summary

Patients with high-risk Ewing sarcoma often have poor outcomes, highlighting the need for better treatment options. This study tested whether giving “window therapy” with irinotecan, temozolomide, and temsirolimus before standard therapy could shrink tumors, and whether patients could tolerate a maintenance phase with sorafenib, bevacizumab, and cyclophosphamide after dose-compressed chemotherapy. Although only three patients met the standard criteria for a treatment response to the window therapy, all showed decreases in tumor size and metabolic activity. Most patients tolerated maintenance therapy well, with only two treatment-related side effects. These results suggest that irinotecan, temozolomide, and temsirolimus demonstrate activity against newly diagnosed Ewing sarcoma, and that maintenance therapy is feasible following aggressive initial treatment. Further research is needed to confirm these findings and improve treatment strategies for this high-risk group.

## 1. Introduction

Ewing sarcoma (ES) is an aggressive small round blue cell tumor most often characterized by EWS–ETS fusions [1]. Current standard care for these patients includes interval compressed chemotherapy, including vincristine, doxorubicin, and cyclophosphamide alternating with ifosfamide and etoposide (VDC/IE), as well as local control (surgery and/or radiation therapy) [1,2]. While 85% of patients initially respond to therapy, approximately 30% of those with localized disease and 70% of those with metastatic disease develop disease-progression or recurrence, with a median time to recurrence of 16 to 21 months [3,4,5]. Additionally, patients with pelvic primary disease or those older than 14 years of age consistently exhibit poorer outcomes [6,7]. Therefore, new treatment strategies that provide both new active agents and approaches that address disease recurrence are needed.

Preclinical and clinical studies support the anti-tumor effects of irinotecan (IRN) and temozolomide (TMZ) in ES [8,9,10,11]. Additionally, ES shows increased expression of mammalian target of rapamycin (mTOR) [12,13], and in preclinical studies, the mTOR inhibitor temsirolimus (TEM) has shown efficacy and synergism when administered with IRN + TMZ. In a phase I study of 71 pediatric patients with solid tumors, IRN + TMZ + TEM (ITT) demonstrated tolerability and a clinical benefit in a subset of patients, including ES [14,15,16,17]. Given the clinical signal of activity and the need for new therapeutic regimens for high-risk ES, the combination of ITT warrants further evaluation.

Over the past decade, maintenance therapy, administered in the setting of minimal residual disease, has garnered increased interest, with the aim of preventing tumor recurrence in multiple cancer types, including pediatric sarcomas. In one study, patients with non-metastatic high-risk rhabdomyosarcoma demonstrated improved survival when maintenance chemotherapy was added to the standard treatment backbone [18]. One strategy to consider for maintenance therapy is to target angiogenesis, which is necessary for tumor growth, metastasis, and survival [19,20,21,22,23]. In a phase I study conducted in pediatric patients with recurrent or refractory solid tumors, low-dose cyclophosphamide (poCY) was combined with the VEGFR and PDGFR-β inhibitor sorafenib (SOR) and the VEGF inhibitor bevacizumab (BEV). In this study, the combination was well tolerated and showed a signal of activity in several tumor types, including ES, providing a rationale for further evaluation [24,25].

We conducted a phase II study for patients with high-risk ES (ESFT13, NCT01946529) to assess the response rate to window therapy (ITT) and the tolerability of maintenance therapy (SOR, BEV, and poCY) added to a standard ES backbone.

## 2. Patients and Methods

### 2.1. Eligibility

Institutional Review Board approval was obtained prior to the study becoming active. Details regarding patient eligibility are defined in the Data Supplement. All patients ≤ 25 years of age with a newly histologically diagnosed ES deemed high-risk were eligible. High-risk was defined as the following: age ≥ 14 years, pelvic primary tumor, or presence of metastatic disease. Informed consent was obtained prior to study enrollment.

### 2.2. Protocol Therapy

#### 2.2.1. Chemotherapy

**Figure 1:**
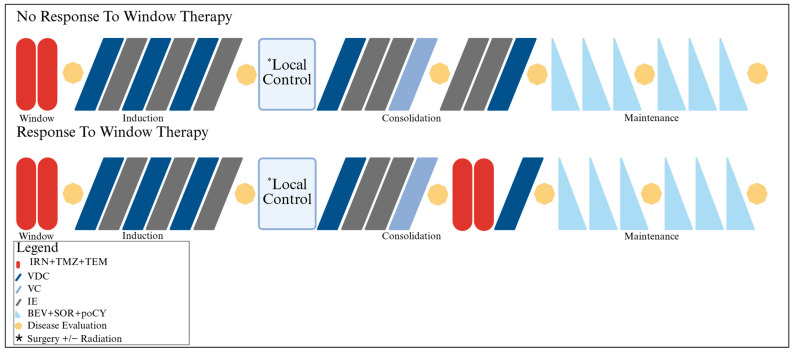
Treatment schema (Created with BioRender.com).

The treatment schema is shown in Figure 1. Participants received window therapy consisting of 2 cycles of IRN (20 mg/m^2^/day IV, days 1–5 and 8–12), TMZ (100 mg/m^2^/day PO, days 1–5), and TEM (35 mg/m^2^/day IV, days 1 and 8) in 21-day intervals. The response was assessed after completion of window therapy, and then patients received a total of 13 cycles of interval-compressed VDC/IE, with local control following cycle 6. Those who had a partial response or better to the window therapy substituted ITT for cycles 11 and 12 of IE. All patients determined to have stable disease or better following induction were eligible to receive maintenance therapy. Maintenance consisted of 6 21-day cycles of BEV (15 mg/kg/dose IV, day 1), SOR (90 mg/m^2^/dose PO, BID days 1–21), and poCY (50 mg/m^2^/dose PO daily, days 1–21).

#### 2.2.2. Local Control

Local control was individually determined in consultation with oncology, surgical, and radiation teams. Approaches included surgery alone, surgery plus radiation, radiation alone, and radiation followed by surgery. The radiation doses are summarized in Supplemental Table S1. Surgery aimed for complete resection with negative margins and functional preservation. Adjuvant radiation was recommended for unresectable tumors, positive margins, or poor histologic response (<90% necrosis).

Metastatic control involved surgery and/or radiation. Radiation was used for the metastatic sites >1 cm not resected with negative margins after induction. All patients with lung metastases at diagnosis received whole-lung radiation; residual unresectable lung lesions >1 cm received a stereotactic body radiation therapy (SBRT) boost.

#### 2.2.3. Toxicity

Toxicity was monitored using the National Cancer Institute Common Terminology Criteria for Adverse Events, version 4.0 (NCI CTCAE v4.0).

#### 2.2.4. Response

All participants who received 2 cycles of window therapy and those with disease-progression were eligible for response assessment. The timing of the tumor evaluations is shown in Figure 1. Details of the response criteria are shown in the Data Supplement. Response to window therapy was assessed using the World Health Organization criteria (WHO). A combination of volumetric response and RECIST 1.1 response was used for the remaining therapy response and for patients not receiving window therapy. Full-body fluorodeoxyglucose-18 positron emission tomography (18F-FDG-PET) was obtained at diagnosis, week 6, week 12, and at the end of treatment to assess the presence of new lesions and to evaluate non-measurable lesions. The peak and mean standard uptake values (SUVpeak and SUVmax) were recorded by an experienced institutional nuclear-medicine physician. A complete list of follow-up evaluations post-therapy is included in Supplemental Table S2.

#### 2.2.5. Quality of Life

Quality of life was assessed using the Peds QL Multidimensional Fatigue Scale, Peds QL Cancer Module, and the Peds QL V4.0 Module. Quality of life measures were obtained for patients at the time of diagnosis (±2 weeks); prior to local control (week 16–19); the end of induction; the end of therapy; and at one, two, five, and ten years off-therapy.

#### 2.2.6. Functional Assessments

The details of the functional assessment are listed in the Data Supplement.

### 2.3. Objectives and Outcomes

The primary objective was to estimate the WHO response (partial response (PR) + complete response (CR)) rate after two initial cycles of window therapy (ITT). Secondary objectives included the following: describing the metabolic response rate, as determined by 18F-FDG PET, associated with window therapy; the toxicities associated with window and maintenance therapy; and the 5-year event-free survival (EFS) and overall survival (OS). The exploratory objectives sought to describe the changes in musculoskeletal health, cardiopulmonary fitness, physical performance, and quality of life in the patients treated within the study.

### 2.4. Statistical Analysis

A two-stage sequential design was used to assess the primary endpoint. If at least 7 of the 12 patients had a tumor response (CR/PR) as defined by WHO criteria, the study would proceed to enroll an additional 11 patients. Otherwise, the study would be closed.

Additional details relating to the statistical analysis are listed in the Data Supplement.

## 3. Results

### 3.1. Patient Characteristics

The patient characteristics are described in Table 1. Seventeen patients were enrolled; one patient declined treatment after enrollment and was excluded from further analysis. Thus, 16 patients were included in the final analysis cohort; all subsequent results pertain to this group. The median age at enrollment was 12.2 years (range: 4.8–23.6), with a near-equal distribution of males (43.8%) and females (56.3%). Most patients self-identified as White (87.5%).

### 3.2. Disease Characteristics

The disease characteristics are shown in Table 1. All but one patient had confirmation of an *EWSR1* translocation by FISH and/or RT-PCR. In the remaining patient, FISH could not be interpreted due to prior decalcification; thus, the diagnosis was established based on morphology and immunohistochemistry. The most common primary site was the axial skeleton (*n* = 10), with six patients having pelvic primaries. Most patients presented with metastatic disease (11/16). Of the additional five patients enrolled, four were included based on age at diagnosis (≥14 years) and one patient was included due to their having a pelvic primary.

### 3.3. Treatment Completed

**Figure 2:**
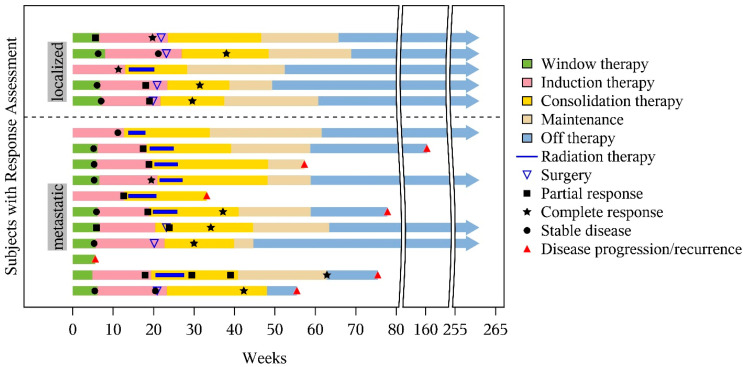
Swimmer plot detailing individual treatments completed and timing of the best responses and follow-up.

Seven of the sixteen patients completed all protocol-directed therapy (including window therapy) and thirteen of the sixteen completed the window therapy (Figure 2). Reasons for not completing the protocol therapy included not being eligible for window therapy (*n* = 3, excluded for requiring emergent radiation), PD (*n* = 1 post window therapy, *n* = 1 during maintenance), toxicity attributed to maintenance therapy (hand–foot skin reaction *n* = 1, immune system disorder *n* = 1), inability to swallow pills (*n* = 1), and physician discretion (*n* = 1).

### 3.4. Tumor Response

**Figure 3:**
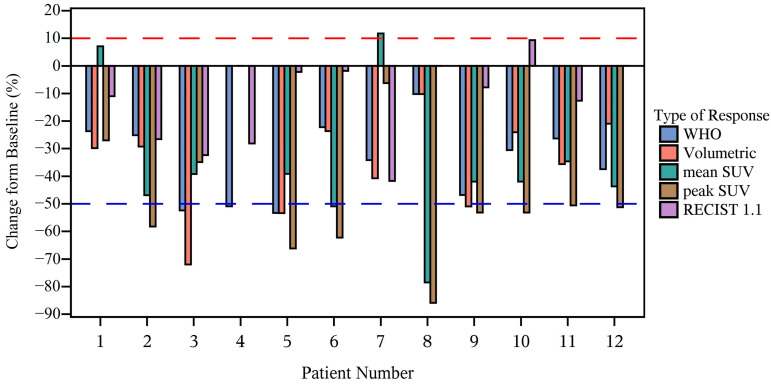
Waterfall plot showing primary tumor response to window therapy according to WHO, RECIST 1.1, PET, and volumetric change. The dashed lines represent the criteria for progressive disease (red) and partial response (blue).

The imaging responses are shown in Figure 3. There were 12/13 patients evaluable for WHO response to the window therapy. One patient did not have disease evaluations performed post window therapy due to physician discretion. Three patients achieved at least a PR by WHO criteria; thus, the trial was stopped for futility. One patient had PD at the completion of window therapy, with the development of a new metastatic lesion. Despite not meeting criteria for PR by WHO, all patients had a statistically significant decline in their SUVmax (mean decline: 33.2%, standard deviation: 26.3%, *p*-value: 0.004) SUVpeak (mean decline: 49.9%, standard deviation: 21.1%, *p*-value: 0.002) and primary tumor volume (mean decline: 32.5%, standard deviation: 17.6%, *p*-value: 0.0005).

### 3.5. Survival Outcomes

**Figure 4:**
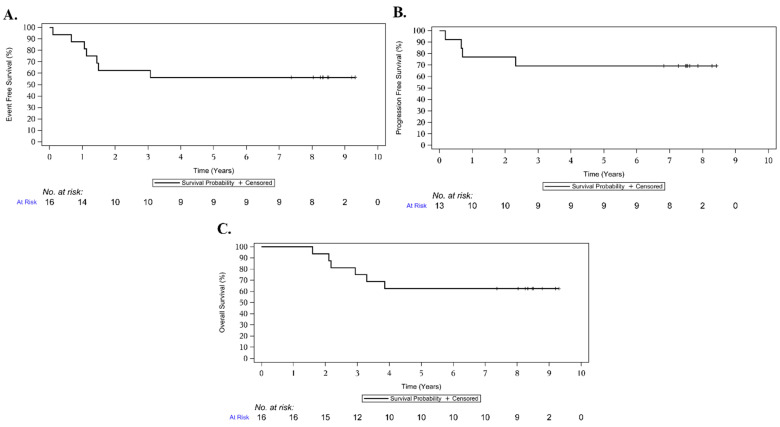
Kaplan–Meier curves with EFS (**A**) and OS (**B**) for all patients treated (*n* = 16) and PFS (**C**) for the patients who started maintenance therapy (*n* = 13).

Kaplan–Meier curves for event-free survival (EFS), overall survival (OS), and progression-free survival (PFS) are shown in Figure 4. Among all treated patients (*n* = 16), the estimated 5-year OS and EFS were 62.5% (95% CI: 34.9, 81.1) and 56.3% (95% CI: 29.5, 76.2), respectively. For the 13 patients who initiated maintenance, the 5-year PFS was 69.2% (95% CI: 37.3, 87.2). Subgroup analyses based on metastatic status, age, and pelvic site are shown in Supplemental Figure S1. While patients with metastatic and pelvic primary trended towards worse outcomes, this trend did not rise to the level of statistical significance for OS. Regarding EFS, only metastatic status reached statistical significance (Log-rank *p* = 0.03, Cox-regression *p* = 0.035).

### 3.6. Primary-Site Local Control and Treatment Failures

Of the fifteen patients eligible for primary-site local control, seven received surgery only, and eight received radiation only. There were no primary-site local failures.

### 3.7. Toxicity

Summaries of treatment-related adverse events are provided in Table 2 and Table 3 and Supplemental Table S3. There were no Grade 4 or greater non-hematologic toxicities reported during window therapy or maintenance therapy.

The most common Grade 3 treatment-related toxicities reported in window therapy were hyponatremia (*n* = 5, 38.5%), colitis (*n* = 4, 30.8%), and diarrhea (*n* = 4, 30.8%).

The most common Grade 3 treatment-related toxicities reported during maintenance therapy were diarrhea (*n* = 2, 15.4%) and palmar–plantar erythrodysesthesia syndrome (*n* = 2, 15.4%). Grade 4 hematologic toxicities were common, including lymphocyte decrease (*n* = 6, 46.2%), neutrophil count decrease (*n* = 4, 30.8%), and white blood cell count decrease (*n* = 3, 23.1%). While only two patients required discontinuation of maintenance therapy due to toxicity, one each with hand–foot skin reaction and immune system disorder, an additional six patients required dose interruptions. The reasons for dose interruptions included neutrophil count decrease (*n* = 3), platelet count decrease (*n* = 1), hematuria (*n* = 1), cystitis (*n* = 1), cardiac disorder (*n* = 1), hand–foot skin reaction syndrome (*n* = 1), and purpura (*n* = 1). One patient required a dose reduction in sorafenib for a hand–foot skin reaction.

### 3.8. Functional Outcomes

Functional assessment outcomes are summarized in Supplemental Figure S2. No statistically significant changes were observed when comparing the results at the end of induction therapy and at the completion of maintenance therapy across the following evaluated domains: Functional Mobility Assessment (FMA, *p* = 1.00), heart rate recovery (HR, *p* = 0.18), grip strength (*p* = 1.00), and maximal oxygen uptake (VO_2_, *p* = 1.00). These findings suggest stability in musculoskeletal and cardiopulmonary performance across the course of protocol therapy.

### 3.9. Quality of Life Outcomes

Individual patient QoL outcomes are shown in Supplemental Figure S3. Combined patient- and parent-reported outcomes are shown in Supplemental Tables S4 and S5. Following initiation of therapy, both patient-reported and parent-reported outcomes showed a trend towards improvement and subsequently remained stable throughout the duration of treatment.

## 4. Discussion

In this phase II trial, the administration of two cycles of window therapy with ITT to children with high-risk ES did not meet the prespecified primary endpoint of a ˃50% WHO objective response rate. However, all but one of the patients demonstrated significant responses according to volumetric and PET assessments, raising concerns about our current methods for evaluating active agents in this disease.

The combination of ITT was well tolerated, with the most common Grade 3 toxicities including diarrhea, colitis, and hyponatremia. There were no unexpected toxicities, and 92% of patients who began window therapy completed protocol therapy through induction. Additionally, patients showed symptomatic improvement that was sustained throughout therapy. The early signals of activity seen on our study reinforce the therapeutic potential of IRN and TMZ in ES, supporting their evaluation in various ongoing and recently completed clinical trials (MSKCC, rEECur, and ONITT) (NCT04901702, NCT01864109, ISRCTN36453794).

Defining the imaging response remains challenging for bone tumors. Both the WHO and the RECIST 1.1 criteria can be applied to bone tumors as target lesions; however, they fail to capture the full extent of the volumetric and metabolic changes occurring, thereby potentially underestimating the early treatment responses [26]. Emerging data suggest that PET response may more accurately correlate with pathologic response in ES [27,28,29], whereas volumetric changes may better predict survival in other sarcoma subtypes [30,31]. In this study, we captured a combination of volumetric, planar, and metabolic responses. Most patients exhibited statistically significant reductions in primary tumor volume, SUVmax, and SUVpeak following window therapy; however, only three proceeded with additional ITT and were defined as “window successes”, due to the protocol-mandated WHO response criteria. Recent data indicate that early disease responses in ES are less predictive of EFS, compared to later responses [32]. This suggests that a study design not requiring early response for treatment continuation may have preserved the accrual, particularly given the encouraging findings from alternative imaging assessments.

Recognizing the limitations of traditional response systems, other disease groups have successfully incorporated criteria that integrate metabolic findings and image-defined risk factors beyond single-plane measurements [33]. Emerging technologies such as machine learning and radiomics also hold promise for improving the predictive value of imaging and strengthening the correlation between early response and long-term outcomes [34,35,36]. Incorporating these modalities into ES evaluation will be critical for the optimization and prioritization of novel agents. Although the use of multiple tumor metrics presents challenges, future ES trials could address these by employing prespecified composite-response definitions that capture volumetric, metabolic, and radiographic changes. Such an approach would better reflect biologically meaningful activity, preserve statistical power in early-phase studies, and reduce the risk of prematurely discarding promising regimens due to the insensitivity of planar criteria alone. Our small sample size limits the ability to draw definitive conclusions regarding certain prognostic factors, such as age and primary tumor location. However, it is noteworthy that none of the patients with localized disease who were over 14 years old or who had primary pelvic tumors experienced an event. The estimated 5-year EFS and OS for the entire cohort were 56.3% (95% CI: 29.5, 76.2) and 62.5% (95 CI: 34.9, 81.1), respectively. Most of our patients (11/16) presented with metastatic disease. Among this subgroup, the estimated 3-year EFS and OS were 45.5% (95% CI: 16.7, 70.7) and 63.6% (95% CI: 29.7, 84.5), with 5-year EFS and OS of 36.4% (95% CI: 11.2, 62.7) and 45.5% (95% CI: 16.7, 70.7), respectively. These outcomes are consistent with recently published metastatic-ES studies [37]. Thus, the presence of metastatic disease at diagnosis and the occurrence of metastatic relapse continue to be the primary reasons for treatment failure in this population, highlighting the potential for the incorporation of maintenance strategies.

A potential barrier to the incorporation of maintenance therapy in the upfront setting in ES has been the concerns relating to tolerability, given the intensity of the upfront treatment. Previous studies that included maintenance therapy were small and used only single agents or alternative treatment backbones [38,39]. In our study, only two of fourteen patients discontinued maintenance due to toxicity, supporting the feasibility of using combination maintenance therapy strategies in future larger, randomized clinical trials. However, the role of maintenance therapy and the optimal regimen still need to be defined.

This study has several important limitations. First, it was a single-center, single-arm phase II trial with a small sample size, which limits generalizability. Second, the planned two-stage design closed at the interim analysis, further reducing statistical power. Finally, the inclusion of patients with metastatic disease, older age, and pelvic primary tumors introduces clinical heterogeneity that may affect comparability across patient populations, particularly as these factors were not controlled for in a randomized design. These limitations underscore the need for confirmation in larger studies of a multicenter nature.

## 5. Conclusions

Our trial demonstrated that ITT is an active combination in ES, despite the study not meeting its primary endpoint. Moreover, it validated the feasibility of combination maintenance therapy following intensive VDC/IE chemotherapy. These results provide a justification for continuing this line of investigation in larger trials of a multicenter nature to more definitively evaluate efficacy and safety. The incorporation of novel imaging and statistical methodologies in future studies could further enhance the precision of response assessment, better capture biologically meaningful activity, and ultimately optimize patient outcomes.

## Figures and Tables

**Table 1:** Summary of demographics and baseline tumor characteristics.

Variables	Freq (%) (*n* = 16)
**Age at Enrollment**	
Median (Min, Max)	12.2 (4.8, 23.6)
**Age Group at Enrollment**	
<14 years	10
≥14 years	6
**Gender**	
Male	7 (43.8)
Female	9 (56.3)
**Race**	
White	14 (87.5)
Multiple Race (NOS)	1 (6.3)
Asian	1 (6.3)
EWSR1 Translocation *	
Positive	15 (93.8)
Negative	1 (6.2)
**Stage**	
Metastatic	11 (68.8)
Localized	5 (31.3)
**Primary Site**	
Extremity	6 (37.5)
Chest Wall	2 (12.5)
Pelvis	6 (37.5)
Spine	1 (6.3)
Renal	1 (6.3)
**Metastatic Site**	
Bone	1 (9.1)
Pulmonary	3 (27.3)
Bone + Bone Marrow	1 (9.1)
Bone + Pulmonary	3 (27.3)
Bone + Bone Marrow + Pulmonary	3 (27.3)
**Primary Tumor Volume**	
*n*	12
Median (Min, Max)	181.3 (12.5, 654.2)

* EWSR1 translocation identified by either FISH or RT-PCR.

**Table 2:** Toxicity during window therapy (*n* = 13).

Category	Grade			
	2 *	**3**	**4**	**5**
**Blood and lymphatic system disorders**				
Anemia	-	3 (23.1%)	-	-
Lymphocyte count decreased	3 (23.1%)	2 (15.4%)	-	-
Neutrophil count decreased	5 (38.5%)	2 (15.4%)	-	-
White blood cell count decreased	3 (23.1%)	-		
**Gastrointestinal disorders**				
Abdominal pain	4 (30.8%)	1 (7.7%)	-	-
Alanine aminotransferase increased	-	1 (7.7%)	-	-
Ascites	-	1 (7.7%)	-	-
Colitis	-	4 (30.8%)	-	-
Diarrhea	4 (30.8%)	4 (30.8%)	-	-
Mucositis oral	-	2 (15.4%)	-	-
Nausea	6 (46.2%)	3 (23.1%)	-	-
Vomiting	4 (80%)	2 (15.4%)	-	-
**General Disorder**				
Fever	2 (15.4%)	-	-	-
**Immune system disorders**				
Allergic reaction	3 (23.1%)	**-**	**-**	**-**
**Infections and infestations**				
Catheter-related infection	-	1 (7.7%)	-	-
Enterocolitis infectious	-	2 (15.4%)	-	-
Esophageal infection	-	1 (7.7%)	-	-
Febrile neutropenia	-	1 (7.7%)	-	-
Mucosal infection	3 (23.1%)	-	-	-
Skin infection	-	1 (7.7%)	-	-
**Metabolism and nutritional disorders**				
Anorexia	8 (61.5%)	2 (15.4%)	-	-
Dehydration	2 (15.4%)	1 (7.7%)	-	-
Hypercalcemia	2 (15.4%)	-	-	-
Hypoalbuminemia	4 (30.8%)	1 (7.7%)	-	-
Hypocalcemia	-	2 (15.4%)	-	-
Hypokalemia	-	2 (15.4%)	-	-
Hyponatremia	-	5 (38.5%)	-	-
Hypophosphatemia	2 (15.4%)	3 (23.1%)	-	-
Weight loss	4 (30.8%)			
**Respiratory, thoracic and mediastinal disorders**				
Pneumothorax	-	1 (7.7%)	-	-
**Maximum Grade Any Event**	3 (23.1%)	10 (76.9%)	-	-

* Only categories with ≥ 10% involvement.

**Table 3:** Toxicity during maintenance therapy (*n* = 13).

Category	Grade			
	2 *	3	4	5
**Blood and lymphatic system disorders**				
Lymphocyte count decreased	-	5 (38.5%)	6 (46.2%)	-
Neutrophil count decreased	-	3 (23.1%)	4 (30.8%)	-
Platelet count decreased	-	2 (15.4%)	-	-
White blood cell count decreased	-	4 (30.8%)	3 (23.1%)	-
**Gastrointestinal disorders**				
Diarrhea	-	2 (15.4%)	-	-
Vomiting	-	1 (7.7%)	-	-
**Infections and infestations**				
Bladder infection	-	1 (7.7%)	-	-
**Renal and urinary disorders**				
Cystitis noninfective	-	1 (7.7%)	-	-
Proteinuria	-	1 (7.7%)	-	-
**Skin and subcutaneous tissue disorders**				
Palmar–plantar erythrodysesthesia syndrome	6 (46.2%)	2 (15.4%)	-	-
**Maximum Grade Any Event**	2 (15.4%)	3 (23.1%)	8 (61.5%)	-

* Only categories with ≥ 10% involvement.

## Data Availability

Data Access: Research data supporting this publication are available upon request to the Corresponding Author, Jessica Gartrell MD, (A) 262 Danny Thomas Place MS 260, Memphis TN 38105, (P) 901-595-3509 (E) jessica.gartrell@stjude.org (F) 901-521-9005.

## Supplementary Materials

The following supporting information can be downloaded at: https://www.mdpi.com/article/10.3390/cancers17172894/s1, Data Supplement: Eligibility criteria, response criteria, functional assessment methods. Figure S1. Kaplan-Meier curves stratified by metastatic status (A,D), pelvic primary (B,E), and age (C,F). Figure S2. Functional outcomes throughout treatment. Figure S3. Quality of life outcomes for each individual patient. Table S1. Summary of radiation therapy doses. Table S2. Post-therapy follow-up evaluations. Table S3. Toxicity during induction therapy (*n* = 15). Table S4. Patient-reported outcomes. Table S5. Parent-reported outcomes. Supplemental References [40,41,42,43,44,45,46,47,48,49,50,51,52,53].
