# Supplementary material for: ESFT13: A Phase II Study Evaluating the Addition of Window and Maintenance Therapy to a Standard Chemotherapy Backbone for the Treatment of High-Risk Ewing Sarcoma
Source: Cancers (Basel). 2025 Sep 3;17(17):2894. doi: 10.3390/cancers17172894 (PMC12428418; doi:10.3390/cancers17172894)
Supplement: Supplementary file 1 [file cancers-17-02894-s001.zip › cancers-3787981-supplementary.pdf]

ESFT13: A Phase II Study Evaluating the Addition of Window and Maintenance Therapy to a Standard Chemotherapy Backbone for the Treatment of High-Risk Ewing Sarcoma

**Data Supplement**


**Page 1. Eligibility Criteria**

**Page 3. Response Criteria**

**Page 7. Functional Assessments**

**Page 10. Statistical Analysis**

**Page 11. References**

**Page 12. Data Supplement Figure Legends**


## Eligibility Criteria

*Inclusion Criteria*

<u>Diagnosis</u>:

Disease must be measurable.

Newly diagnosed patient with histologically proven ESFT involving the bone or soft tissue and at least one of the following:

- Metastatic disease (must be biopsy proven*)
- Pelvic primary
- Age $\geq$ 14 years at the time of diagnosis

*Patients with more than one pulmonary lesion >1cm may be considered as having evidence of metastatic disease without biopsy, as long as there is no other clear medical reason for these lesions. In cases where there was doubt, biopsy was encouraged.

Age: < 25 years

Adequate organ function defined as:

- a peripheral absolute neutrophil count (ANC) > 750/m3 and platelet count > 75,000/m3 (no transfusion within 7 days of enrollment). Patients with EST metastatic to the bone marrow were not required to meet bone marrow criteria for study eligibility and were not evaluable for hematologic toxicity.
- Creatinine clearance or radioisotope GFR ≥ 50 ml/min/1.73m$^2$ (GFR  40 ml/min/1.73m2 if < 2 years of age).
- total bilirubin < 3.0 mg/dL, SGOT (AST) or SGPT (ALT) ≤ 3.0 x ULN for age
- shortening fraction > 28%.

Reproductive Status: Females of childbearing potential and males able to father a child had to be

willing to practice acceptable methods of birth control to prevent pregnancy.

Other

No CYP3A4 inducers or inhibitors within 1 week prior to study entry

Serum triglyceride level ≤ 300mg/dL and serum cholesterol ≤ 300 mg/dL

Random fasting glucose with the upper limits of normal. If random was abnormal, fasting glucose had to be within normal range

*Exclusion Criteria*

Patient pregnant or breastfeeding

Inability or unwillingness to give written informed consent

Prior history of malignancy with the exception of non-melanoma skin cancer

## Response Criteria

*Response to Window Therapy*

Tumor response to window therapy was assessed after two cycles of ITT using similar response criteria as outlined in POG 9457 (WHO criteria). The criteria are

outlined below:

- Complete response (CR): No evidence of disease
- Clinical complete response (CCR): Ninety percent decrease in the sum of the products of the maximum perpendicular diameters of up to five of the largest measurable metastatic soft tissue lesion(s) and improvement in bone scan/PETCT or bony architecture.
- Partial response (PR): Greater than or equal to 50% decrease in the sum of the products of the maximum perpendicular diameters of up to five of the largest measurable metastatic soft tissue lesion(s). No evidence of progression in any lesions, no new lesions.
- No response (NR): Less than 50% decrease in the sum of the products of the maximum perpendicular diameters of up to five of the largest measurable metastatic soft tissue lesion(s).
- Progressive disease (PD): Greater than or equal to 25% increase in the sum of the products of the maximum perpendicular diameters of up to five of the largest measurable metastatic soft tissue lesion(s).

*Overall Response*

All research participants with measurable primary tumor (a lesion that could accurately be measured in at least one dimension with the diameter at least twice the thickness of the C or MRI slice) had volumetric measurements of the primary tumor using an elliptical model (0.5 times the product of the three largest perpendicular diameters) to assess response to neo-adjuvant therapy. The Response Evaluation

Criteria in Solid Tumors (RECIST) version 1.1 from the NCI (http://ctep.cancer.gov/guidelines/templates.html) was used for assessment of metastases.

Primary tumor response was defined as below:

- *Complete response (CR):* complete disappearance of the tumor
- *Partial response (PR):* at least 50% decrease in volume compared to the measurement obtained at study entry
- *Progressive disease (PD):* at least 25% increase in tumor volume compared to the smallest volume obtained since the beginning of therapy
- *Stable disease (SD):* Neither sufficient shrinkage to qualify for PR nor sufficient increase to qualify for PD taking as a reference the smallest disease volume since treatment started

Metastatic tumor response was defined as below:

*Target lesions*:

- *Complete response (CR):* Disappearance of all target lesions. Any pathological lymph nodes (whether target or non-target) must have a reduction is short axis to < 10 mm. If immunocytology is available, no disease must be detected by that methodology.
- *Partial response (PR):* At least a 30% decrease in the disease measurement, taking as reference the disease measurement done to confirm measurable disease at study entry.
- *Progressive disease (PD):* At least a 20% increase in the disease measurement, taking as reference the smallest disease measurement recorded since the start of treatment; or the appearance of one or more new lesions. In addition to the relative increase of 20% the sum must also demonstrate an absolute increase of at least 5 mm. (Note: the appearance of one or more new lesions is also considered progression).
- *Stable disease (SD):* Neither sufficient shrinkage to qualify for PR nor sufficient increase to qualify for PD taking as reference the smallest disease measurement since the treatment started.

*Non-target lesions:*

- *Complete response (CR):* Disappearance of all nontarget lesions. All lymph nodes must be < 10 mm in short axis.
- *Non-complete response/Non-progressive disease (Non-PD*): Persistence of one or more non-target lesion(s).
- *Progressive disease (PD):* Appearance of one or more new lesions and/or unequivocal progression of existing non-target lesions. Note: Unequivocal progression of non-target lesions implies that the patient has PD overall.

<u>Overall Response</u>

| **Primary Tumor*** | **Target Metastatic Lesions** | **Non-target Metastatic Lesions** | **New lesions** | **Overall Response** |
|---|---|---|---|---|
| CR | CR | CR | No | CR |
| CR | CR | Incomplete response/Non-PD | No | PR |
| CR | PR | Non-PD | No | PR |
| PR | CR/PR/SD | Non-PD | No | PR |
| SD | CR/PR | Non-PD | No | PR |
| SD | SD | Non-PD | No | SD |
| PD | Any | Any | Yes or No | PD |
| | PD | Any | Yes or No | PD |
| | | | | PD |

| | | | | |
|---|---|---|---|---|
| Any | Any | PD | Yes or No | PD |
| Any | Any | Any | Yes | |
| Any | | | | |

CR: complete response; PR: partial response; SD: stable disease; PD: progressive disease

*If CR was not attained because of a residual primary tumor which was then completely resected at second look surgery, the participant was considered as conversion to CR status by surgery.

<u>Response criteria with non-measurable disease only</u>:

- *Objective response*: All of the following criteria must be met to qualify as an objective response:
    - Decrease in avidity or number of lesions if initially abnormal on bone scan, FDG PET, CT or MRI
    - MRI: Decrease in the size or number of bone lesion/s. Bone Marrow: Tumor cell burden must decrease to 50% of baseline.
- *No response*: All the following criteria must be met to qualify as a no response:
    - No new lesions on bone scan, FDG PET, CT or MRI
    - MRI: No increase in the size of bone lesions
    - Bone marrow: Tumor cell burden < 50% decreased or < 25% increased from baseline.
- *Progressive disease*: Appearance of new areas of disease will qualify for progressive disease. For pre-existing lesions, all the following criteria must be met:
    - MRI: increase in the size of the lesions

- Bone marrow: increase in tumor cell burden 25% from baseline.

**Functional Assessments**

Functional assessments were obtained at the time of diagnosis (± 2 weeks), prior to local control (week 16-19), end of induction, end of therapy, and 12 months off therapy.

*Range of Motion (ROM)*

Active and passive range of motion measurements of the surgical and nonsurgical-extremities (for limb tumors) or appropriate axial body part (neck/trunk/jaw) were obtained with a goniometer. Impairment was defined as >20% deficit in the involved joint compared to age and sex specific normative values[1-3].

*Muscular Strength*

Muscular strength was evaluated by maximum value of measured isometric shoulder flexion or maximum isometric knee extension (Biodex System III), depending on tumor location. Impairment was defined as the involved joint's strength being < 90% of uninvolved joint's strength[4]. If involved and uninvolved sides could not be defined due to tumor location, then the maximum measured bilateral value was comparted to age and sex related normative values[5-7]. Impairment was defined as measured strength < 90% of normative values[4].

*Grip strength*

Grip strength was measured with a hand held dynamometer (Jamar), and the maximum of four measurements (two on each hand) was used for analysis. Impairment was defined as measured grip strength < one standard deviation below the mean of age and sex related normative values[8,9].

*Functional Mobility Assessment (FMA)*

The FMA is a reliable and valid tool to assess a patient's objective functional abilities. A FMA was performed in patients 4 years and older. The FMA requires patients to physically perform specific tasks, which can objectively quantify their level of function. The FMA is comprised of six subcategories: 1) Pain, 2) Function with two specific measures, Timed Up and Down Stairs (TUDS) time and Timed Up and Go (TUG) time. Heart rate and rate of perceived exertion are measured during the TUDS and TUG, 3) Supports, 4) Satisfaction with walking quality, 5) Participation in work, school, sports, and 6) Endurance as measured by the 9-minute run-walk test. Physiological cost index (PCI), heart rate, and rate of perceived exertion (RPE) are also measured during the 9-minute run-walk.

For the TUDS portion, patients walked up and down 12 stairs. A stopwatch was used to record the time in seconds. For the TUG patients had to stand up from sitting in a chair, walk 3 meters, turn around, return to the chair, and sit down.120,123 A stopwatch was used to record the time in seconds.

For the Nine-minute run walk the patient was instructed to run or walk as far as possible in 9 minutes.124 Patients were allowed to walk, but their objective was to cover as much distance as possible in the time allowed. A wheeled feet counter was used to measure the distance traveled to the nearest foot. The patients were asked their RPE immediately after the TUG, TUDS, and 9-minute run walk test. The PCI was determined at the completion of the 9-minute run walk test. Heart rate was measured with a heart rate monitor while the patient performed the TUDS, TUG, and 9-minute run walk at each time the RPE and PCI were obtained.

Pain was measured by using a numerical or FACES scale. For patients 8 years of age or older, the medical staff person/therapist asked patients if they have experienced any pain in the past week and were presented with a pain scale in which 0 represents no pain and 10 represents the worst pain imaginable. For patients 4 to 7 years of age, pain was measured by using the FACES scale. The Wong-Baker FACES pain rating scale consists of a series of six faces (from a very happy face, representing no pain to a very sad face, representing the worst pain imaginable) with corresponding numbers from 0 to 10.

Supports, Satisfaction, and Participation were measured by a series of questions pertaining to pain, supports (brace, cane and crutches), satisfaction with walking ability, and participation in sports, work and school in relation to the whole person and specific to the lower-extremities. The scores were based on a 0 to 5 scale.

Each question and test were totaled. Impairment was defined as a score in the lowest 10% of the age and sex specific normative range[10].

*Heart Rate (HR)*

A resting ECG was completed to examine heart rate, rhythm, hypertrophy, and ischemia to rule out any acute cardiac problems prior to exercise testing. A standard 12 lead electrocardiogram (the Med Graphics Cardio Perfect® Resting/Stress ECG system) was collected for 15 seconds at 25mm/s with a gain setting of 10 mm/V with the patient in a recumbent position for 10 minutes. HR was considered impaired if it was one or more standard deviations below age and sex specific normative mean value [11].

*Vo2*

Maximal cardiopulmonary exercise testing with ECG and breath by breath gas exchange analysis was completed on a treadmill or cycle ergometer (available in both upper and lower extremity models) while using an incremental ramping protocol. During this test, ECG, and gas exchange was monitored continuously. Exercise testing continued until exhaustion, or one of the following signs/symptoms:

- A decrease in ventricular rate with increasing workload associated with symptoms of inadequate cardiac output.
- Failure of heart rate to increase with exercise and symptoms indicating inadequate cardiac output.
- Progressive fall in systolic blood pressure with increasing workload
- Severe hypertension in excess of 250mmHg systolic or 125 mmHg diastolic
- Dyspnea the participant finds intolerable
- Symptomatic tachycardia the participant finds intolerable

- Presence of a ≥ 3 mm flat or downward sloping ST-segment depression
- Increasing ventricular ectopy with increasing workload, including a > 3- beat run
- Patient requests termination of the study.

Predicted cardiopulmonary fitness (VO-2) was calculated based on either the FRIEND equation (age: ≥ 18 years), or the pediatric equation from James et al. (< 18 years)[12,13]. The pediatric equation is additionally adjusted for body surface area which was calculated using the Dubois method[14]. Measured $VO_2$ from the cardiopulmonary exercise test (CPET) was divided by predicted $VO_2$ to calculated percent predicted. Quantiles were calculated to determine the 50$^{th}$ percentile of the cohort's percent predicted. Any patient below the 50$^{th}$ percentile (54.3% of predicted) was considered impaired.

**Statistical Analysis**

Descriptive statistics were used to summarize patient and disease characteristics. Means and medians were reported for continuous variables and frequencies and percentages reported for categorical variables. The Wilcoxon signed-rank tests were used to test the percent changes in tumor responses (WHO, RECIST 1.1, tumor volume, and PET parameters) after the window therapy relative to initial evaluations. The Kaplan-Meier estimates were reported for 5-year overall survival (OS), event-free survival (EFS), and progression-free survival (PFS). For the purposes of EFS and PFS, an event was defined as death, disease progression, recurrence, or second malignancy. EFS was defined as the initiation of protocol directed therapy to the time of event or loss to follow-up. PFS was defined as the time from the initiation of maintenance therapy to the time of event or loss to follow-up. OS was defined as the initiation of the therapy to the time of death or loss to follow-up.

The effects from risk factors-- such as age at enrollment (age < 14 and age ≥14), metastatic status (localized and metastatic) and primary tumor site (pelvic and non-pelvic) -- on OS, EFS and PFS were tested using the log-rank tests. The hazard ratios and the corresponding 95% confidence intervals for these risk factors were estimated using the Cox regressions with Firth's correction. The statistical

significance level was set at a p-value of 0.05 for all analyses in this study. All statistical analyses were conducted using the software SAS 9.4 and R 4.2.1. software

For MSK objectives the Wilcoxon signed-rank tests were used to compare impairment assessment results between baselines and time points across the study (prior to local control, end of induction, end of treatment, and follow-up month 12) for functional mobility assessment (FMA), range of motion (ROM), grip, strength, heart rate and VO2.

## References


1. Moromizato, K.; Kimura, R.; Fukase, H.; Yamaguchi, K.; Ishida, H. Whole-body patterns of the range of joint motion in young adults: masculine type and feminine type. *J Physiol Anthropol* **2016**, *35*, 23, doi:10.1186/s40101-016-0112-8.
2. Soucie, J.M.; Wang, C.; Forsyth, A.; Funk, S.; Denny, M.; Roach, K.E.; Boone, D. Range of motion measurements: reference values and a database for comparison studies. *Haemophilia* **2011**, *17*, 500-507, doi:10.1111/j.1365-2516.2010.02399.x.
3. Troke, M.; Moore, A.P.; Maillardet, F.J.; Hough, A.; Cheek, E. A new, comprehensive normative database of lumbar spine ranges of motion. *Clin Rehabil* **2001**, *15*, 371-379, doi:10.1191/026921501678310171.
4. Neeter, C.; Gustavsson, A.; Thomeé, P.; Augustsson, J.; Thomeé, R.; Karlsson, J. Development of a strength test battery for evaluating leg muscle power after anterior cruciate ligament injury and reconstruction. *Knee Surg Sports Traumatol Arthrosc* **2006**, *14*, 571-580, doi:10.1007/s00167-006-0040-y.
5. Harbo, T.; Brincks, J.; Andersen, H. Maximal isokinetic and isometric muscle strength of major muscle groups related to age, body mass, height, and sex in 178 healthy subjects. *Eur J Appl Physiol* **2012**, *112*, 267-275, doi:10.1007/s00421-011-1975-3.
6. Holm, I.; Fredriksen, P.; Fosdahl, M.; Vøllestad, N. A normative sample of isotonic and isokinetic muscle strength measurements in children 7 to 12 years of age. *Acta Paediatr* **2008**, *97*, 602-607, doi:10.1111/j.1651-2227.2008.00709.x.
7. K.A. Khalaf, M.P. A normative database of isokinetic upper-extremity joint strengths: towards the evaluation of dynamic human performance. *Biomedical Engineering: Applications, Basis and Communications* **2001**, *13*, 79-92.
8. Günther, C.M.; Bürger, A.; Rickert, M.; Crispin, A.; Schulz, C.U. Grip strength in healthy caucasian adults: reference values. *J Hand Surg Am* **2008**, *33*, 558-565, doi:10.1016/j.jhsa.2008.01.008.
9. Mathiowetz, V.; Wiemer, D.M.; Federman, S.M. Grip and pinch strength: norms for 6- to 19-year-olds. *Am J Occup Ther* **1986**, *40*, 705-711, doi:10.5014/ajot.40.10.705.
10. Marchese, V.G.; Oriel, K.N.; Fry, J.A.; Kovacs, J.L.; Weaver, R.L.; Reilly, M.M.; Ginsberg, J.P. Development of reference values for the Functional Mobility Assessment. *Pediatr Phys Ther* **2012**, *24*, 224-230, doi:10.1097/PEP.0b013e31825c87e7.

11. Fleming, S.; Thompson, M.; Stevens, R.; Heneghan, C.; Plüddemann, A.; Maconochie, I.; Tarassenko, L.; Mant, D. Normal ranges of heart rate and respiratory rate in children from birth to 18 years of age: a systematic review of observational studies. *Lancet (London, England)* **2011**, *377*, 1011-1018, doi:10.1016/s0140-6736(10)62226-x.
12. James, F.W.; Kaplan, S.; Glueck, C.J.; Tsay, J.Y.; Knight, M.J.; Sarwar, C.J. Responses of normal children and young adults to controlled bicycle exercise. *Circulation* **1980**, *61*, 902-912, doi:10.1161/01.cir.61.5.902.
13. Myers, J.; Kaminsky, L.A.; Lima, R.; Christle, J.W.; Ashley, E.; Arena, R. A Reference Equation for Normal Standards for VO(2) Max: Analysis from the Fitness Registry and the Importance of Exercise National Database (FRIEND Registry). *Prog Cardiovasc Dis* **2017**, *60*, 21-29, doi:10.1016/j.pcad.2017.03.002.
14. Shuter, B.; Aslani, A. Body surface area: Du Bois and Du Bois revisited. *Eur J Appl Physiol* **2000**, *82*, 250-254, doi:10.1007/s004210050679.

## Data Supplement Figure Legends

Figure S1: Kaplan-Meier curves stratified by metastatic status (A,D), pelvic primary (B,E), and Age (C,F)

Figure S2: Functional outcomes throughout treatment

Figure S3: Quality of life outcomes by each individual patient

**Table S1.** Summary of radiation therapy doses.

| **Summary of radiation therapy doses** | | |
|---|---|---|
| | Volume dose (cGy) | Cumulative dose (cGy) |
| **Definitive radiotherapy** | | |
| *Primary tumor <8cm* | | |
| PTV1 (GTV1 + CTV1) | 4500 | 4500 |
| PTV2 (GTV2) | 1080 | 5580 |
| *Primary tumor ≥ 8cm* | | |
| PTV1 (GTV1 + CTV1) | 4500 | 4500 |
| PTV2 (GTV2) | 1980 | 6480 |
| **Post-operative radiotherapy** | | |
| PTV1 (GTV1 + CTV1) | 5040 | 5040 |
| **Post-operative brachytherapy and external beam radiotherapy** | | |
| CTV1b (brachytherapy HDR) | 1360 (4 fxn BID) | 1360 |
| PTB1 (GTV1+CTV1+CTV1b) | 4500 | 5860 |
| **Post-operative brachytherapy** | | |
| CTV1b | 3400 (10 fxn BID) | 3400 |
| **Metastatic Site** | | |
| Hypofraction (preferred) | 3000 | 3000 |
| Standard fractionation | 5400 | 5400 |
| **Special metastatic site** | | |
| Whole lung irradiation-PTV Lung | 1650 | 1650 |
| Pleural surface irradiation- PTV Pleura | 3000 | 2400-3000 |
| Whole abdomen irradiation- PTV Abdomen | 3000 | 3000 |

cGy: centigray; PTV: primary tumor volume; GTV1; gross tumor volume; CTV1: clinical target volume

**Table S2.** Post-therapy follow-up evaluations

| Studies to be obtained (Approximate months) after EOT | 3 | 6 | 9 | 12 | 15 | 18 | 21 | 24 | 30 | 36 | 42 | 48 | 52 | 60 | 72-120 |
|---|---|---|---|---|---|---|---|---|---|---|---|---|---|---|---|
| **Clinical and Laboratory** | | | | | | | | | | | | | | | |
| History | X | X | X | X | X | X | X | X | X | X | X | X | X | X | X[6] |
| Physical exam (height, weight, BSA, vital signs | X | X | X | X | X | X | X | X | X | X | X | X | X | X | X[6] |
| CBC, differential, platelets | X | X | X | X | X | X | X | X | X | X | X | X | X | X | X[6] |
| Urinalysis | X | X | X | X | X | X | X | X | X | X | X | X | X | X | X[6] |
| Serum chemistries[1] | X | X | X | X | X | X | X | X | X | X | X | X | X | X | X[6] |
| **Diagnostic Imaging** | | | | | | | | | | | | | | | |
| DEXA | X | | | | | | | | | | | | | X | |
| Body FDG PET and/or bone scan[2] | | X | | X | | X | | X | | | | | | | |
| CT chest | X | X | X | X | X | X | X | X | X | X | X | X | X | X | |
| Plain AP bilateral knees Group B only[3] | | | | X | | | | | | | | | | | |
| MRI primary tumor site (non-limb sparing only) | X | X | X | X | X | X | X | X | X | X | X | X | X | X | |
| XR primary tumor (extremity only) | X | X | X | X | X | X | X | X | X | X | X | X | X | X | |
| **Endocrine and Fertility** | | | | | | | | | | | | | | | |
| LH, FSH, estradiol, Anti-mullerian hormone (AMH) (females) | | X | | X | | | | X | | X | | X | | X | X |

| **Studies to be obtained (Approximate months) after EOT** | **3** | **6** | **9** | **12** | **15** | **18** | **21** | **24** | **30** | **36** | **42** | **48** | **52** | **60** | **72-120** |
|---|---|---|---|---|---|---|---|---|---|---|---|---|---|---|---|
| LH, FSH, testosterone (free & total), inhibin B (males) | | X | | X | | | | X | | X | | X | | X | X |
| Semen analysis (males) | | | | X | | | | | | X | | | | X | X |
| TSH, total and free T4, reverse and total T3 (Group B only)[4] | X | X | | X | | | | | | | | | | | |
| Tanner Staging by endocrinologist | | | | X | | | | X | | X | | X | | X | X |
| **Cardiac and Pulmonary Monitoring** | | | | | | | | | | | | | | | |
| Troponin T, NT-pro BNP | | | | X | | | | X | | X | | X | | X | X[6] |
| ECG/ECHO | | | | X | | | | X | | X | | X | | X | X[6] |
| Pulmonary function test[5] | | | | X | | | | | | X | | | | X | X |
| **Functional Outcomes and Health-Related QoL** | | | | | | | | | | | | | | | |
| Functional outcomes | | | | X | | | | X | | | | | | X | X |
| QoL Measures | | | | X | | | | X | | | | | | X | X |

[1]Chemistries include Na, K, Cl, CO2, glucose, calcium, magnesium, phosphorous, blood urea nitrogen, creatinine, SGPT, SGOT, total bilirubin, alkaline phosphatase, albumin, total protein, lactate dehydrogenase, uric acid
[2]Determination of whether a PETCT and/or bone scan should be performed should be based on whether initial tumor was detectable by one or either imaging study.
[3]Plain AP bilateral knees only in participants who received maintenance therapy and had open growth plate at the end of treatment.
[4]Obtain only in participants who received maintenance therapy.
[5]Obtain at 12, 36, 60 and once between 96 – 120 months only in participants who had primary chest wall tumor or lung metastases at diagnosis.
[6]Recommended yearly.

**Table S3.** Toxicity During Induction Therapy (N=15)

| Category | Grade | | | |
|---|---|---|---|---|
| | **2*** | **3** | **4** | **5** |
| **Blood and lymphatic system disorders** | | | | |
| Anemia | - | 10 (66.7%) | 5 (33.3%) | - |
| Lymphocyte count decreased | - | - | 15 (100%) | - |
| Neutrophil count decreased | - | 1 (6.7%) | 14 (93.3%) | - |
| Platelet count decreased | - | 3 (20%) | 12 (80%) | - |
| White blood cell decreased | - | 1 (6.7%) | 14 (93.3%) | - |
| **Gastrointestinal disorders** | | | | |
| Alanine aminotransferase increased | - | 3 (20%) | - | - |
| Aspartate aminotransferase increased | - | 1 (6.7%) | - | - |
| Colitis | - | 2 (13.3%) | - | - |
| Diarrhea | - | 1 (6.7%) | - | - |
| Esophagitis | - | 1 (6.7%) | - | - |
| Lipase increased | - | 1 (6.7%) | - | - |
| Mucositis oral | - | 5 (33.3%) | - | - |
| Nausea | - | 2 (13.3%) | - | - |
| Oral pain | - | 1 (6.7%) | - | - |
| Vomiting | - | 2 (13.3%) | - | - |
| **General disorders and administration site conditions** | | | | |
| Non-cardiac chest pain | - | 1 (6.7%) | - | - |
| **Infections and infestations** | | | | |
| Anorectal infection | - | 1 (6.7%) | - | - |
| Catheter-related infection | - | 2 (13.3%) | - | - |
| Enterocolitis infectious | - | 1 (6.7%) | - | - |
| Esophageal infection | - | 2 (13.3%) | - | - |
| Febrile neutropenia | - | 13 (86.7%) | - | - |
| Infections and infestations - Other, specify | - | 1 (6.7%) | - | - |
| Lung infection | - | 2 (13.3%) | - | - |
| Mucosal infection | 2 (13.3%) | 1 (6.7%) | - | - |

| Category | Grade | | | |
| --- | --- | --- | --- | --- |
| | **2*** | **3** | **4** | **5** |
| | - | 1 (6.7%) | - | - |
| Otitis media | | | | |
| Vaginal infection | - | 1 (6.7%) | - | - |
| Vulval infection | - | 1 (6.7%) | - | - |
| Wound infection | - | 1 (6.7%) | - | - |
| **Metabolism and nutrition disorders** | | | | |
| Hypocalcemia | - | 1 (6.7%) | - | - |
| Hypokalemia | - | 5 (33.3%) | - | - |
| **Vascular disorders** | | | | |
| Hypotension | - | 1 (6.7%) | - | - |
| **Maximum Grade Any Adverse Event** | | | | |
| Number of Patients | - | - | 15 (100%) | - |

*Only categories with ≥ 10% involvement

**Table S4.** Patient reported outcomes

| Variables | Time* | | | | | | |
|---|---|---|---|---|---|---|---|
| | **1** | **2** | **3** | **4** | **5** | **6** | **7** |
| **Psychosocial** | | | | | | | |
| N | 15 | 14 | 14 | 14 | 6 | 5 | 4 |
| Median (Min, Max) | 71.7 (37.5, 80.0) | 76.7 (61.4, 95.0) | 77.5 (56.7, 96.7) | 78.8 (40.0, 96.7) | 84.2 (70.0, 100.0) | 90.0 (63.3, 100.0) | 78.3 (65.0, 96.7) |
| Mean (SD) | 65.8 (13.0) | 78.0 (10.4) | 78.4 (13.0) | 77.0 (16.8) | 83.9 (12.0) | 86.3 (14.6) | 79.6 (13.3) |
| **Physical** | | | | | | | |
| N | 15 | 14 | 14 | 14 | 6 | 5 | 4 |
| Median (Min, Max) | 52.2 (15.6, 81.3) | 51.6 (21.9, 93.8) | 60.0 (18.8, 93.8) | 73.4 (21.9, 95.7) | 87.5 (37.5, 100.0) | 90.6 (21.9, 95.7) | 93.8 (68.8, 93.8) |
| Mean (SD) | 50.5 (23.9) | 56.5 (23.0) | 60.9 (25.4) | 65.1 (22.8) | 80.5 (23.1) | 77.9 (31.5) | 87.5 (12.5) |
| **Total** | | | | | | | |
| N | 15 | 14 | 14 | 14 | 6 | 5 | 4 |
| Median (Min, Max) | 57.6 (30.7, 78.3) | 72.8 (50.0, 88.0) | 77.2 (43.5, 91.7) | 75.5 (31.9, 95.7) | 85.3 (59.8, 98.9) | 89.1 (48.9, 97.8) | 83.7 (66.3, 95.7) |
| Mean (SD) | 59.5 (16.3) | 70.2 (12.4) | 71.7 (15.7) | 71.1 (18.4) | 83.2 (14.7) | 83.3 (19.9) | 82.3 (12.2) |
| **Fatigue** | | | | | | | |
| N | 15 | 14 | 14 | 13 | 6 | 5 | 5 |
| Median (Min, Max) | 62.5 (34.7, 88.9) | 60.4 (38.9, 100.0) | 74.3 (33.3, 97.2) | 72.2 (23.6, 97.2) | 81.9 (55.6, 100.0) | 75.0 (47.2, 100.0) | 66.7 (52.8, 97.2) |
| Mean (SD) | 60.4 (15.8) | 65.7 (17.3) | 69.6 (17.6) | 70.9 (20.0) | 79.4 (18.5) | 74.4 (19.6) | 73.3 (21.9) |
| **Symptom** | | | | | | | |
| N | 14 | 14 | 14 | 13 | 6 | 5 | 5 |
| Median (Min, Max) | 69.6 (30.6, 87.0) | 71.8 (55.6, 94.2) | 75.9 (53.7, 98.1) | 78.8 (40.7, 94.4) | 92.1 (76.9, 99.1) | 80.6 (75.9, 100.0) | 86.5 (76.0, 99.1) |
| Mean (SD) | 64.2 (16.8) | 74.8 (11.9) | 77.5 (13.3) | 75.7 (14.9) | 89.8 (9.2) | 84.5 (10.1) | 88.2 (8.8) |

*Time = Treatment time point
1 = Pre-study; 2 = Prior to Local Control; 3 = End of Induction; 4 = End of Therapy; 5 = 1 Year off Therapy; 6 = 2 Years off Therapy; 7 = 5 Years off Therapy

**Table S5.** Parent reported outcomes

| Variables | Time* | | | | | | |
|---|---|---|---|---|---|---|---|
| | **1** | **2** | **3** | **4** | **5** | **6** | **7** |
| **Parent_Psychosocial** | | | | | | | |
| N | 16 | 15 | 15 | 15 | 6 | 5 | 4 |
| Median (Min, Max) | 70.8 (35.0, 96.7) | 72.5 (41.7, 90.0) | 80.0 (38.6, 95.0) | 75.0 (60.0, 96.2) | 82.7 (70.0, 100.0) | 86.7 (76.7, 100.0) | 98.3 (88.3, 100.0) |
| Mean (SD) | 66.4 (19.3) | 72.8 (15.5) | 73.7 (17.1) | 77.2 (13.1) | 84.5 (12.8) | 88.0 (10.2) | 96.2 (5.5) |
| **Parent_Physical** | | | | | | | |
| N | 16 | 15 | 15 | 15 | 6 | 5 | 4 |
| Median (Min, Max) | 39.1 (0.0, 93.8) | 53.1 (15.6, 93.8) | 68.8 (15.6, 90.6) | 62.5 (31.3, 96.9) | 84.4 (18.8, 96.9) | 93.8 (75.0, 100.0) | 92.2 (68.8, 100.0) |
| Mean (SD) | 41.1 (29.4) | 54.8 (25.2) | 61.5 (23.0) | 61.5 (20.4) | 76.0 (29.4) | 88.1 (10.9) | 88.3 (14.1) |
| **Parent_Total** | | | | | | | |
| N | 16 | 15 | 15 | 15 | 6 | 5 | 4 |
| Median (Min, Max) | 58.8 (25.0, 89.1) | 69.6 (38.8, 84.8) | 76.1 (31.6, 87.0) | 71.7 (51.1, 93.8) | 83.1 (52.2, 98.9) | 86.1 (76.1, 97.8) | 96.2 (81.5, 100.0) |
| Mean (SD) | 57.3 (20.3) | 66.3 (15.1) | 69.2 (17.7) | 71.7 (14.6) | 81.5 (17.2) | 88.3 (9.4) | 93.5 (8.2) |
| **Parent_Symptom** | | | | | | | |
| N | 8 | 8 | 8 | 8 | 4 | 3 | 3 |
| Median (Min, Max) | 67.1 (21.2, 86.5) | 75.9 (65.4, 90.4) | 77.8 (51.0, 93.0) | 76.4 (53.8, 96.3) | 83.9 (67.3, 97.2) | 98.1 (84.6, 100.0) | 93.8 (91.3, 100.0) |
| Mean (SD) | 61.2 (21.2) | 78.1 (9.3) | 76.0 (14.2) | 74.4 (17.5) | 83.1 (13.2) | 94.3 (8.4) | 95.0 (4.5) |

* Time = Treatment time point

1 = Pre-study; 2 = Prior to Local Control; 3 = End of Induction; 4 = End of Therapy; 5 = 1 Year off Therapy; 6 = 2 Years off Therapy; 7 = 5 Years off Therapy

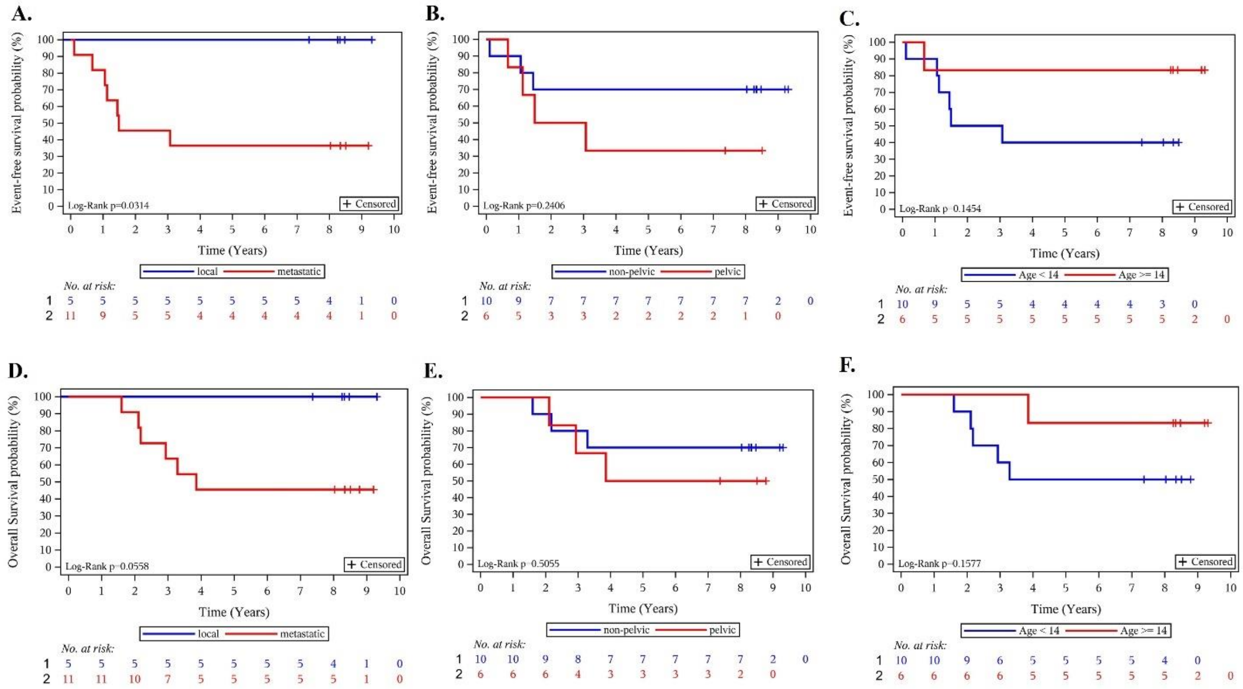


Figure S1: Kaplan-Meier curves stratified by metastatic status (A, D), pelvic primary (B, E), and Age (C, F)

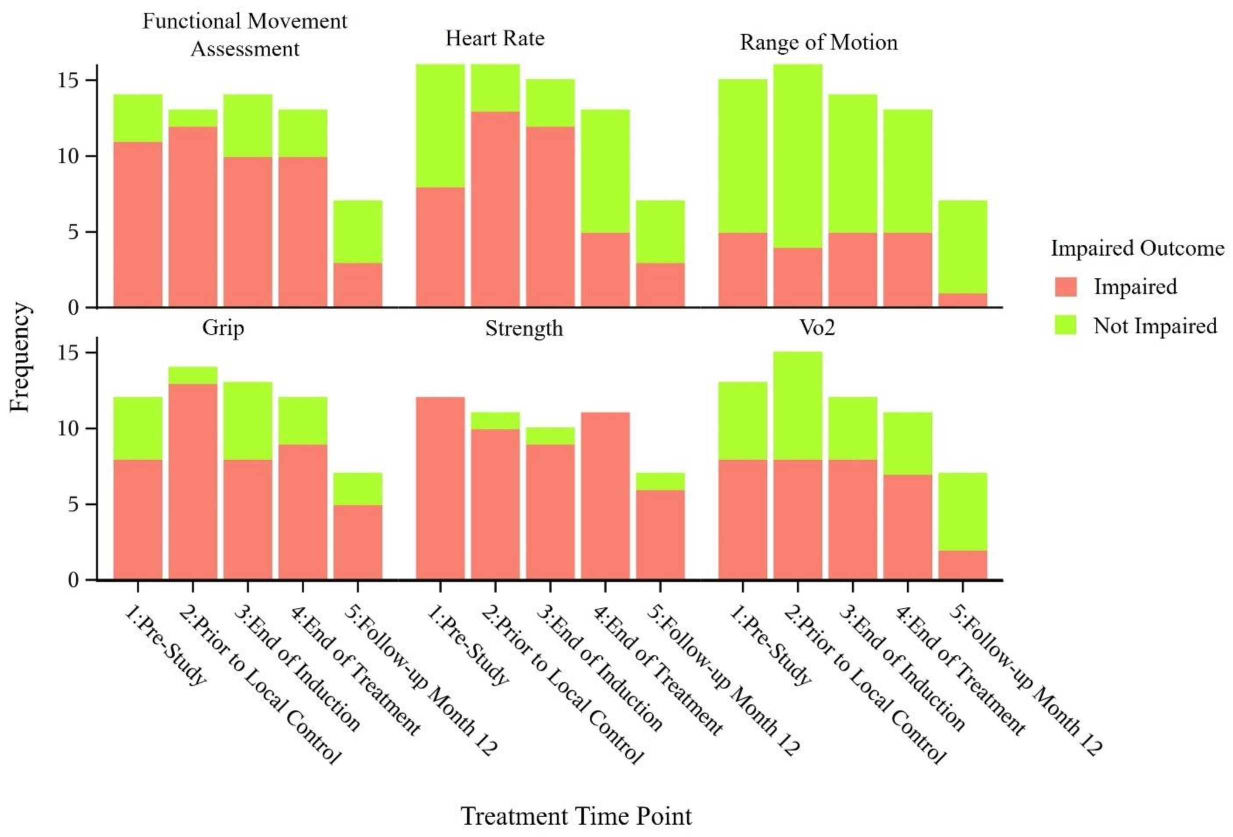


Figure S2. Functional outcomes throughout treatment

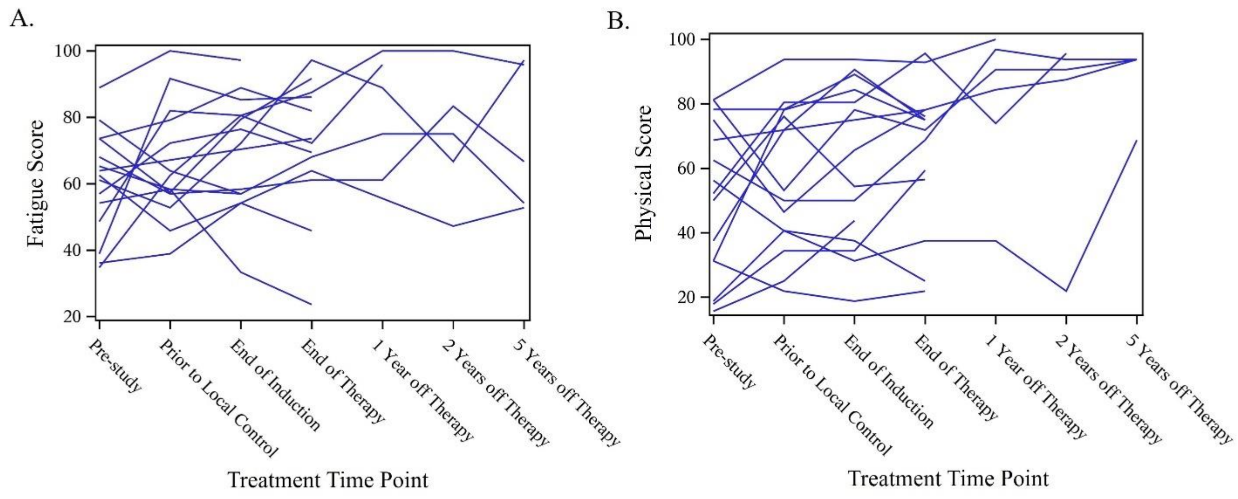


Figure S3: Quality of life outcomes by each individual patient
